# Association between Internet Addiction and Application Usage among Junior High School Students: A Field Survey

**DOI:** 10.3390/ijerph18094844

**Published:** 2021-05-01

**Authors:** Kentaro Kawabe, Fumie Horiuchi, Rie Hosokawa, Kiwamu Nakachi, Shu-ichi Ueno

**Affiliations:** 1 Center for Child Health, Behavior and Development, Ehime University Hospital, Shitsukawa, Toon-City 791-0295, Japan; kawabe.kentaro.fj@ehime-u.ac.jp (K.K.); dcoca14tf@gmail.com (R.H.); waffle9315@yahoo.co.jp (K.N.); 2 Department of Neuropsychiatry, Ehime University Graduate School of Medicine, Ehime University Hospital, Shitsukawa, Toon-City 791-0295, Japan; ueno@m.ehime-u.ac.jp

**Keywords:** application, gaming disorder, internet addiction, junior high school, pathological Internet use

## Abstract

The purpose of this study was to elucidate the relationship between the severity of Internet addiction and various media-related applications. The participants were junior high school students between 12 and 15 years old. A total of 529 students (283 males, 246 females) were included. The participants answered Young’s Internet Addiction Test (IAT) and a structural questionnaire about their access to electronic devices and applications. An Internet addiction prevalence of 4.3% (95% CI: 2.8–6.5%) was reported in this study, with an additional 26.3% (95% CI: 22.6–30.2%) of participants possibly addicted. The accessibility of gaming devices was significantly higher in male students than in female students. The use of applications for SNSs was significantly higher in female students than in male students. Twitter accessibility was a factor that contributed to Internet addiction in both genders. The prevalence of severe Internet addiction among school students in Japan was 4.3%, and Twitter was the most important factor associated with this addiction. Media literacy must be increased in adolescents and their friends, teachers, and families.

## 1. Introduction

Recently, there have been increasing concerns about gaming disorder (GD), smartphone addiction, and social network service (SNS) fatigue caused by Internet addiction. In particular, GD was officially included in the World Health Organization’s International Classification of Diseases (ICD-11) in 2019 [1]. Although not formally recognized as a diagnosis, research into SNS addiction has expanded over recent years [2]. Internet addiction is associated with various psychosocial issues, and psychological issues or a stressful event may play a role in the etiology of Internet addiction [3]. In general, adolescents have been found to spend more time on the Internet than adults, predisposing themselves to Internet addiction. Internet addiction and smartphone addiction can cause serious behavioral addictions and hikikomori in Japanese young adults [4]. The high prevalence of Internet addiction reflects a common and problematic issue worldwide [5].

A previous study found that virtual communications on the Internet rather than social interactions in real life could lead to maladaptation [6]. However, the Internet is currently used to provide a greater variety of and more interesting virtual communications such as SNS or online gaming to alleviate negative feelings. Sometimes, these various media devices and applications, or psychological issues themselves, may cause Internet addiction. Thus, it is important to identify the effects of media devices and psychological issues in adolescents with Internet addiction.

In our previous study [7], we demonstrated that higher addiction levels were associated with severe mental problems and smartphones were most closely associated with Internet addiction in Japanese junior high school students (odds ratio (OR): 1.72, 95% confidence interval (CI): 1.23–2.40). However, to our knowledge, no study has investigated which media devices or smartphone applications affect Internet addiction. The purpose of the present study was to investigate the prevalence of Internet addiction among junior high school students, to elucidate the relationship between Internet addiction and various media devices or smartphone applications.

## 2. Materials and Methods

### 2.1. The Study Design

The data for this study were obtained from a cross-sectional study conducted in November 2018 in Masaki town (population: 30,700) in the suburban area of Japan. There are three public junior high schools in the survey area, but only two were included in this study. The study included all junior high school students from these two schools attending school on the day of this survey, excluding students in special needs education class between 12 and 15 years old. Prior to the data collection, all participants were informed of the purpose, procedure, and confidentiality of the study in detail. The participants were given paper-based anonymous questionnaires in the classroom. A total of 529 students were included in the analysis (proper response rate, 98.0%). Ten students who did not participate and one who provided an inappropriate response were excluded. The Institutional Review Board of Ehime University Graduate School of Medicine approved the present study (IRB No. 1809010), and all participants signed the informed consent in accordance with the Declaration of Helsinki.

### 2.2. Instruments

The participants responded to questions regarding their age, gender, and weekday and weekend Internet usage time and to a structural questionnaire about electronic devices. In addition, the participants completed Young’s Internet Addiction Test (IAT) [8].

The IAT is one of the most frequently used self-administered instruments to investigate Internet addiction [9]. The IAT consists of 20 items and is calibrated with scores ranging from 1 to 5 (given a total score ranging from 20 to 100), with higher scores reflecting a greater tendency toward addiction [8]. The definition of electronic devices in IAT include all electronic devices with an Internet connection, such as laptop computers, mobile phones, smartphones, and gaming consoles. Three types of Internet user groups were identified in accordance with the original scheme of Young [10]. Respondents with IAT scores higher than 70 were classified as “Addicted” and most likely have encountered significant life problems due to excessive Internet use. Respondents with IAT scores between 40 and 69 were classified as “Possibly addicted”. Respondents with IAT scores below 39 were classified as “Not addicted” and are most likely average Internet users who only have some problems controlling their Internet use. A previous study reported that academic performance was negatively correlated with the Internet addiction scores using the psychometric properties and factor structure of the IAT [11]. In the present study sample (*n* = 529), the Cronbach’s α coefficient was 0.937.

The participants completed a structural questionnaire regarding their exposure or access to electronic devices, such as televisions, laptop computers, tablet computers, smartphones, portable games (e.g., Nintendo DS and PlayStation Vita), and console games (e.g., Nintendo Switch, PlayStation 4, and Xbox) as well as applications on tablet devices (e.g., Line and Twitter). The participants were asked to respond with a “yes” or “no” to these questions.

### 2.3. Data Collection Procedure

The study protocol was explained to the junior high school teachers of the area. The school teachers explained the purpose of the present study to the students, and they completed the questionnaires during class using a self-report, anonymous format. The students’ primary caregivers were informed of this study via a letter and were asked to contact the school for clarification.

### 2.4. Statistical Analyses

The study results were expressed as means (±SD) for continuous variables and as percentages for categorical variables. Descriptive statistics were used to describe the distributions of the participants’ characteristics. Chi-square tests were employed to compare the use of electronic devices between genders. Logistic regression models were used to assess the independent relationships between Internet addiction and the accessibility of certain electronic devices. Based on the IAT scores, we reclassified the participants into two groups: the non-addicted group (IAT scores < 39) and the Internet addiction group (IAT scores > 40). We performed a series of multivariable logistic stepwise regression analyses individually. All tests were two sided, and the significance level was 5%. All data were analyzed using SPSS Statistics software version 22.0 (IBM Corp., Armonk, NY, USA) for Windows and R version 3.6.3.

### 2.5. Procedure

Junior high school teachers were informed about the aims and methods of this study by the authors, and parents were informed through a letter along with instructions to contact the school for further clarification if needed. In November 2018, homeroom teachers explained the purpose of the study to their students, who then filled in the questionnaires during class using a self-reporting format. About 20 min were allotted for students to complete the questionnaires. As the questionnaires were anonymous, individual results were not reported to each student. Students were informed that they could freely choose whether or not to participate in this study and were reassured that their choice would not affect their school life in any way.

## 3. Results

### 3.1. Characteristics of the Study Population

The study sample consisted of 529 (283 males, 246 females) junior high school students, with an average age of 13.7 ± 0.9 years. A total of 4.3% (23/529, 95% CI: 2.8–6.5%) of the participants were addicted, with addiction prevalence of 4.9% (14/283, 95% CI: 2.7%–8.2%) in males and 3.7% (9/246, 95% CI: 1.7–6.8%) in females. Additionally, 26.3% (139/529, 95% CI: 22.6–30.2%) of the participants were possibly addicted, including 24.7% (70/283, 95% CI: 19.8–30.2%) of males and 28.0% (69/246, 95% CI: 22.5–34.1%) of females. Table 1 presents a summary of the sociodemographic characteristics and time spent online divided into three groups based on IAT scores. There were significant differences between grades and the time spent online on weekdays and on weekends (*p* < 0.001). The average time spent on the Internet on weekdays was 114.2 ± 113.8 min among first-grade students, 131.4 ± 100.1 min among second-grade students, and 149.4 ± 138.0 min among third-grade students. The average time spent on the Internet on weekends was 237.2 ± 202.1 min among first-grade students, 252.0 ± 178.8 min among second-grade students, and 280.9 ± 208.3 min among third-grade students. No significant differences were found according to gender.

### 3.2. Characteristics of Students According to Accessibility of Electronic Devices and Applications Used

Table 2 shows the number and proportion of students who had free access to each electronic device and the commonly used applications. The accessibility of laptop computers was significantly lower in male students than in female students. The accessibility of console games and portable games was significantly higher in male students than in female students. The accessibility of smartphones did not differ significantly according to gender.

### 3.3. Formatting of Mathematical Components

The use of applications with online videos (e.g., YouTube) exceeded 85% in all students. Less than 5% of the participants used Facebook or did not have access to the Internet. The use of applications about music, e-books, mobile learning, LINE messenger, and social media networks, such as Twitter, Instagram, Facebook, and TikTok, was significantly higher in female students than in male students. The use of gaming applications was significantly higher in male students than in female students.

### 3.4. Factors That Contribute to Internet Addiction

Table 3 shows the results of the multivariable logistic regression analysis of the factors related to Internet addiction among the male and female students, independently. Those variables of electronic devices and application that had usage rates under 10% or above 90% were removed. The other variables underwent multivariable logistic stepwise regression analyses with the Internet addiction of students as the dependent variable. The Hosmer–Lemeshow test revealed *p*-values of 0.997 and 0.714 for males and females, respectively, indicating that the model adequately fit the data. The entire model explained between 8.4% (Cox and Snell R2 = 0.084) and 11.9% (Nagelkerke R2 = 0.119) of the variance of Internet addiction in males and between 22.9% (Cox and Snell R2 = 0.229) and 32.1% (Nagelkerke R2 = 0.321) of the variance of Internet addiction in females.

As shown in Table 3, we found that the accessibility of Line messenger (OR: 3.07, 95% CI: 1.56–6.08; *p* = 0.001) and Twitter (OR: 1.90, 95% CI: 1.01–3.56; *p* = 0.045) contributed to Internet addiction in males. In females, we found that the accessibility of Twitter (OR: 3.98, 95% CI: 2.16–7.31; *p* < 0.001), e-books (OR: 3.46, 95% CI: 1.60–7.49; *p* = 0.002), and videos on demand (OR: 3.35, 95% CI: 1.63–6.85; *p* = 0.001) contributed to Internet addiction.

## 4. Discussion

This was the first study to investigate the prevalence of Internet addiction among junior high school students in Japan. In the current study (*n* = 529), 26.3% of participants were possibly addicted to the Internet and 4.3% were addicted. The risk factors of Internet addiction included Twitter in both genders, LINE messenger in males, and videos on demand and e-books in females.

A previous meta-analysis reported that the most frequently used scale was the IAT, and the prevalence rates of Internet addiction in teenagers ranged from 1.6% to 20.6% [5]. These differences in prevalence were due to diverse study designs, different assessment methods, different social and cultural contexts, sampling from different subpopulations, and the timing of the investigations [12]. A characteristic point in our study was the narrow age range of 12 to 15 years old, because the participants included only junior high school students. Our findings revealed that the prevalence of Internet addiction and time spent online differed by age and grade. In Japan, almost all third-grade students in junior high school must take entrance examinations for senior high school. In addition, the current study was conducted during the examination season for third-grade students. The different levels of academic pressure and the availability of leisure time in each grade may explain the varying risk of Internet addiction across different grades [13]. Significantly more time was spent online in both the addicted and possibly addicted groups than in the non-addicted group on both weekdays and holidays. The more students use the Internet, the greater the risk of Internet addiction [14].

Some studies have reported that males are at risk of higher IAT scores than females [5,15]; however, according to this study, no gender difference was shown regarding the prevalence of addiction according to IAT scores. Our study showed that males used games more frequently than females, whereas females used SNSs more frequently. The results of this study were similar to another study in college students [16]. Tateno et al. reported [17] that 1.7% of college students were Facebook users and 36.3% were Twitter users, indicating a similar tendency to this study. These results also showed that Facebook is rarely used among teenagers in Japan.

Our previous study regarding Internet addiction among adolescents and indicates that participants with Internet addiction have a propensity to have peer problems [7]. Peer relationships are the most important predicting factor of Internet addiction [18]. Virtual life on the Internet is more attractive because it allows users to escape their problems in real life [19]. A previous study reported that the positive qualities of social networking can be helpful for adolescents’ development by maintaining and enhancing communication with peers whom they know in everyday contexts and might mitigate the risk of Internet addiction [20]. In addition, individuals could alleviate their negative feelings by using the Internet when facing stressors from negative life situations [14]. However, SNSs have shown that adolescents can suffer from negative psychological consequences when using these sites excessively [21]. A previous study reported that loneliness is related to Internet addiction [22]. Lonely and depressed individuals prefer online social interactions to face-to-face interactions, which may lead to compulsive Internet use and psychosocial problems associated with Internet use [23]. Adolescents who do not gain support from their parents and friends will gradually lose their self-esteem. Perceived social support from parents and friends can increase self-esteem in adolescents with Internet addiction [22]. Thus, school-aged students with Internet addiction must receive social support from their supporters including their friends, teachers, families, etc.

It is important to determine an effective intervention target in terms of electronic devices and applications to prevent Internet addiction. Based on the logistic regression analyses, the accessibility of Twitter, among other devices and applications evaluated, was found to be the greatest contributing factor to Internet addiction in the study population. The constant development of online social media-related services has increased the number of social media users of LINE, Instagram, and TikTok; however, many users have deviated themselves from social media use due to social media fatigue [24,25]. Wartberg et al. reported that one in three adolescents with a problematic social media use was affected by depressive symptoms [26]. In 6-month follow-up study, social media use was independently associated with the development of depression among young adults [27]. Social media dependence was more prominent in teenagers and young adults, and they more likely to use of Twitter over Instagram and Facebook [28]. Twitter is one of the most popular SNSs in the world. Twitter allows users to read and post short messages with a maximum of 140 characters. According to the company Twitter Japan, there are over 326 million active Twitter users in the world, with 45 million active users in Japan in 2018 [29]. In addition, Japan displayed the greatest rate of increase of Twitter users globally in 2015 [29]. However, several serious issues regarding Twitter use have been widely reported in Japan, including issues related to the leakage of personal information, kidnapping, or running away from home, sexual issues in underage persons, and murder cases. Therefore, The Cabinet Office in Japan has begun to provide information on the proper usage of Twitter in underage individuals. Internet addiction principally involving Twitter is associated with some psychological factors, such as loneliness and social interaction skills [30]. Persons who are lonely tend to prefer socializing on the Internet, which leads to the overuse of social networking sites [31].

There are several limitations in our study. First, this study used a cross-sectional design; therefore, it cannot determine cause-and-effect relationships, as can longitudinal studies. Second, we did not control for confounding factors such as economic status and academic performance. Third, the results are limited because of the small sample size of participants who used some devices and applications.

## 5. Conclusions

The study found a high prevalence of Internet addiction among school students in Japan, and males used games more frequently, whereas females used SNSs more frequently. Twitter use was the most important factor associated with Internet addiction in students both male and female. These findings suggest the presence of harmful effects of problematic Internet use in the study setting. Parents should know their children’s online activities and gain digital literacy. From an early prevention perspective, the present findings also suggest the need the digital literacy and safety skills for children in future research. The proper use of Internet and the knowledge of the Internet addiction should be necessary in students, their parents, and school teachers.

## Figures and Tables

**Table 1:** Demographic characteristics of the participants.

	Total	Not Addicted	Possibly Addicted	Addicted	φ	*p*
N (%)	529 (100)	367 (69.4)	139 (26.3)	23 (4.3)		
IAT scores	36.0 ± 15.2	27.8 ± 5.9	50.5 ± 8.7	79.9 ± 6.8		
Gender, *n* (%)					0.046	0.568
Male	283 (100)	199 (70.3)	70 (24.7)	14 (4.9)		
Female	246 (100)	168 (68.3)	69 (28.0)	9 (3.7)		
Grade, *n* (%)					0.196	<0.001
First	175 (100)	136 (77.7)	35 (20.0)	4 (2.3)		
Second	179 (100)	131 (73.2)	39 (21.8)	9 (5.0)		
Third	175 (100)	100 (57.1)	65 (37.1)	10 (5.7)		
					**F**	***p***
Age, year	13.7 ± 0.9	13.6 ± 0.9	13.8 ± 1.0	14.0 ± 0.7	3.9	0.021
Time spent online						
Weekday	131.6 ± 118.8	112.0 ± 112.8	166.2 ± 110.0	234.8 ± 159.4	20.8	<0.001 ^a^
Weekend	256.6 ± 197.1	215.3 ± 169.5	324.2 ± 196.0	500.9 ± 302.0	38.5	<0.001 ^a^

IAT: Young’s Internet Addiction Test. ^a^ Addicted > Possibly addicted > Not addicted.

**Table 2:** Characteristics of students according to accessibility of electronic devices and applications used.

Electronic Devices N = 529	All *n* (%)	Males *n* (%)	Females *n* (%)	χ	*p* Value
Television	441 (83.4)	231 (81.6)	210 (85.4)	1.328	0.249
Laptop computer	187 (35.3)	89 (31.4)	98 (39.7)	4.052	0.044 *
Tablet computer	241 (45.6)	124 (43.8)	117 (47.4)	0.744	0.388
Smartphone	341 (64.5)	176 (62.2)	165 (66.8)	1.369	0.242
Portable game	381 (72.0)	220 (77.7)	161 (65.2)	9.867	0.002 **
Console game	348 (65.8)	203 (71.7)	145 (58.7)	9.562	0.002 **
Application					
Online videos (e.g., YouTube)	466 (88.1)	255 (90.1)	211 (85.8)	2.356	0.125
Music	353 (66.7)	165 (58.3)	188 (76.4)	19.460	<0.001 **
Gaming application	331 (58.8)	180 (63.6)	131 (53.3)	5.821	0.016 *
Video on demand	90 (17.0)	43 (15.2)	47 (19.1)	1.426	0.232
LINE messenger	394 (74.5)	190 (67.1)	204 (82.9)	17.261	<0.001 **
Twitter	138 (26.1)	57 (20.1)	81 (32.9)	11.157	0.001 **
Instagram	93 (17.6)	25 (8.8)	68 (27.6)	32.130	<0.001 **
TikTok	221 (41.8)	89 (31.4)	132 (53.6)	26.688	<0.001 **
Facebook	17 (3.2)	5 (1.8)	12 (4.9)	4.096	0.043 *
E-book	64 (12.1)	25 (8.8)	39 (15.9)	6.098	0.014 *
Flea market applications (e.g., Mercari, Rakuma)	73 (13.8)	34 (12.0)	39 (15.9)	1.631	0.202
Web browsing	93 (17.6)	45 (15.9)	48 (19.5)	1.184	0.276
Mobile learning	239 (45.2)	116 (41.0)	123 (50.0)	4.314	0.038 *
No internet use	16 (3.0)	9 (3.2)	7 (2.8)	0.050	0.823

Chi-square tests. * *p* < 0.05, ** *p* < 0.01.

**Table 3:** Multiple logistic regression analysis of effect of electronic devices and applications on internet addiction.

	Male Total *n* = 283	Male IA *n* = 84	Adjusted	Female Total *n* = 246	Female IA *n* = 78	Adjusted
Electronic devices	N (%)	N (%)	OR (95%CI)	N (%)	N (%)	OR (95%CI)
Laptop computer	89 (31.4)	34 (40.5)		98 (39.7)	35 (44.9)	
Tablet computer	124 (43.8)	40 (47.6)		117 (47.4)	42 (53.8)	
Smartphone	176 (62.2)	57 (67.9)		165 (66.8)	60 (76.9)	
Portable game	220 (77.7)	64 (76.2)		161 (65.2)	54 (69.2)	
Console game	203 (71.7)	64 (76.2)		145 (58.7)	50 (64.1)	
Music	165 (58.3)	52 (61.9)		188 (76.4)	59 (75.6)	
Gaming application	180 (63.6)	63 (75.0)		131 (53.3)	52 (66.7)	
Video on demand	43 (15.2)	19 (22.6)		47 (19.1)	26 (33.3)	3.35 (1.63–6.85) **
LINE messenger	190 (67.1)	71 (84.5)	3.07 (1.56–6.08) **	204 (82.9)	72 (92.3)	
Twitter	57 (20.1)	27 (32.1)	1.90 (1.01–3.56) *	81 (32.9)	44 (56.4)	3.98 (2.16–7.31) **
Instagram	25 (8.8)	11 (13.1)		68 (27.6)	27 (34.6)	
TikTok	89 (31.4)	37 (44.0)		132 (53.6)	44 (56.4)	
E-book	25 (8.8)	10 (11.9)		39 (15.9)	23 (29.5)	3.46 (1.60–7.49) **
Flea market applications	34 (12.0)	16 (19.0)		39 (15.9)	22 (28.2)	
Web browsing	45 (15.9)	16 (19.0)		48 (19.5)	23 (29.5)	
Mobile learning	116 (41.0)	43 (51.2)		123 (50.0)	44 (56.4)	

Internet addiction: addicted and possibly addicted. OR: odds ratio, CI: confidence interval adjusted for other factors in multiple logistic regression analyses with stepwise elimination procedure at the *p* = 0.05 significance level for entry into the model. * *p* < 0.05, ** *p* < 0.01. Cox and Snell R2 = 0.084; Nagelkerke R2 = 0.119 (male). Cox and Snell R2 = 0.229; Nagelkerke R2 = 0.321 (female).
